# Functional Connectivity States of Alpha Rhythm Sources in the Human Cortex at Rest: Implications for Real-Time Brain State Dependent EEG-TMS

**DOI:** 10.3390/brainsci12030348

**Published:** 2022-03-03

**Authors:** Davide Tabarelli, Arianna Brancaccio, Christoph Zrenner, Paolo Belardinelli

**Affiliations:** 1 Center for Mind/Brain Sciences—CIMeC, University of Trento, I-38123 Trento, Italy; davide.tabarelli@unitn.it (D.T.); arianna.brancaccio@unitn.it (A.B.); 2 Temerty Centre for Therapeutic Brain Intervention, Centre for Addiction and Mental Health, Department of Psychiatry, University of Toronto, Toronto, ON M6J 1H4, Canada; christoph.zrenner@gmail.com; 3 Department of Neurology & Stroke, University of Tübingen, D-72070 Tübingen, Germany

**Keywords:** alpha oscillations, functional connectivity, source reconstruction, MEG, EEG state-dependent TMS

## Abstract

Alpha is the predominant rhythm of the human electroencephalogram, but its function, multiple generators and functional coupling patterns are still relatively unknown. In this regard, alpha connectivity patterns can change between different cortical generators depending on the status of the brain. Therefore, in the light of the communication through coherence framework, an alpha functional network depends on the functional coupling patterns in a determined state. This notion has a relevance for brain-state dependent EEG-TMS because, beyond the local state, a network connectivity overview at rest could provide further and more comprehensive information for the definition of ‘instantaneous state’ at the stimulation moment, rather than just the local state around the stimulation site. For this reason, we studied functional coupling at rest in 203 healthy subjects with MEG data. Sensor signals were source localized and connectivity was studied at the Individual Alpha Frequency (IAF) between three different cortical areas (occipital, parietal and prefrontal). Two different and complementary phase-coherence metrices were used. Our results show a consistent connectivity between parietal and prefrontal regions whereas occipito-prefrontal connectivity is less marked and occipito-parietal connectivity is extremely low, despite physical closeness. We consider our results a relevant add-on for informed, individualized real-time brain state dependent stimulation, with possible contributions to novel, personalized non-invasive therapeutic approaches.

## 1. Introduction

The origin of alpha waves and the function they subserve constitute long-lasting scientific issues in neuroscience. Already by 1929, Berger had managed to isolate alpha waves by means of a pioneering EEG set-up using scalp electrodes and described this rhythm as the most prominent in the human electroencephalogram [1]. Recent advances due to brain source reconstruction and invasive electrophysiological recordings have provided evidence that alpha waves originate from several cortical and subcortical sites, with direct evidence suggesting both thalamus and diverse cortical areas as possible origins of such rhythm [2,3,4].

The functional role of alpha oscillations also is still far from being completely understood. The “pulsed inhibition hypothesis”, for instance, proposes that alpha oscillations actively inhibit neuronal firing in a phasic manner, opening and closing interleaved periods of “high” and “low” excitability of the cortex by cyclically producing bouts of inhibition [5,6]. This hypothesis is in line with the idea behind brain-state dependent stimulation, that the outcome of an electro/magnetic perturbation depends on the instantaneous phase state of a specific brain rhythm in a given area. In fact, studies investigating alpha in the occipital cortex demonstrate that the alpha phase at the instant of a visual stimulus predicts high or low probability of detection [7].

However, it is still not clear which is the exact role of alpha oscillations in opening states of cortical excitability. For example, the aforementioned “pulsed inhibition hypothesis” does not seem to apply to findings of brain-state dependent stimulation in the parietal cortex, where the same directionality of inhibition vs. excitation as in the visual cortex does not emerge [8,9,10]. In this regard, studies investigating µ-alpha in the parietal cortex show that a principle of pulsed facilitation rather than inhibition would better explain the role of alpha in the hand-knob of the sensorimotor cortex. In fact, it has been shown by means of real-time EEG phase-dependent transcranial magnetic stimulation (TMS) that stimulation at µ-alpha troughs results in facilitated motor evoked responses (MEPs, [8]), while at peaks of the same oscillation, inhibition does not seem to occur in a significant proportion of instances [9].

Even if there is no consensus as to whether alpha shapes neuronal recruitment by determining windows of lower and higher excitability, or rather only by opening windows of greater excitability, alpha oscillations appear to have a role in shaping neural recruitment.

Most of the protocols conceived to modulate cortical excitability by means of TMS use a predefined stimulus sequence irrespective of the instantaneous brain-state, as opposed to a real-time brain-state dependent stimulation, which delivers the stimulus in determined phases of the alpha cycle [8]. If the phase of alpha reflects a phasic increase and decrease of cortical excitability (as reported in the occipital cortex by [11], we should find different outcomes depending on the local alpha phase at the instant of stimulation. First attempts in this direction have tried to post-hoc determine the phase of alpha waves at the moment of the stimulus. This has been mainly tested in the occipital cortex, where the conscious visual percept has been linked post-hoc to the alpha phase at the instant of stimulus presentation [7,12]). However, the effects were generally assessed by statistically estimating the probability that the stimulus could be delivered at a given phase. Differently, phase-state dependent stimulation leverages instantaneous EEG phase-states to trigger the TMS pulse exactly at the phase of interest, without post-hoc tracing of it back to the moment of analysis. Therefore, studies using this novel approach have led to more consistent and reproducible results. For example, EEG-TMS has been used to investigate motor excitability depending on mu-alpha in the parietal cortex. This line of research is relatively recent, but several consistent pieces of evidence show that participants with a detectable mu-alpha rhythm show larger motor evoked potentials (MEPs) when TMS stimulation at the motor spot is delivered at mu troughs or at the early rising phase, compared to the conditions where positive peaks or random phases are targeted [8,13,14]. These results are in line with the hypothesis of the alpha rhythm as a mechanism modulating cortical excitability. Attempts to link specific alpha phases to enhanced cortical excitability have also been made in studies targeting the dorsolateral frontal cortex (DLPFC), where alpha-synchronized rTMS at troughs appears to provide for a local alpha power decrease in patients with drug resistant depression disorder. The same result is not obtained by intermittent theta burst or random phase stimulation [15].

To sum up, the role of alpha phase has been investigated in at least three different cortical areas and a relationship between alpha phase and cortical excitability has been found in frontal, parietal and occipital regions [8,11,16]. However, there is still no clear and organic consensus on a generalized functional role of the alpha phase. For example, the alpha phase in the occipital cortex seems to elicit opposite effects with respect to the other cortical areas: trough targeting has been proposed to decrease the possibility of perceiving the stimulus [7]. Moreover, it has been shown that the conscious perception of a visual stimulus not only depends on alpha phase in the occipital cortex but also on that in fronto-central regions at the moment of stimulation [7,12] this would be in line with the idea of the frontal control network allowing access to a conscious perception.

This evidence, together with the assumption that coherence between brain regions underlies integration of information [17], suggests that knowing the functional coupling between stimulated (or post-hoc investigated) regions and other areas potentially connected with the stimulation site is crucial in designing experiments aiming at a trial-based selective perturbation of a given “brain state”. In this light, considering the functional coupling patterns of the stimulated site is important in explaining EEG-TMS results and is even more crucial when EEG-TMS protocols are intended, not only in terms of a single stimulation site, but also as a technique for pathway-specific modulation targeting multiple functional hubs: the next horizon of brain-state dependent stimulation. In this regard, this technique has been shown to have the potential to modulate specific pathways [18], for example, by coupling the stimulation to an activity state of that pathway (e.g., [13]). Therefore, reliable metrics are required that can extract extended network states through long-range connectivity. Such solid brain state landmarks would be certainly useful (before and after a neuro-modulatory intervention) to assess whether the brain-stimulation has exerted the desired network state modulation. Most importantly, however, they would be even more crucial when time-resolved connectivity state estimates, describing the connectivity pattern of the network, are used as real-time trigger condition. Nevertheless, gold-standard metrics for connectivity generally consist of pseudo-statistics regarding different phase consistency over several tens of trials and a consensus for a conceptual definition of single-trial instantaneous connectivity is still missing. For this reason, we here try to open the way to addressing the need for brain-state dependent protocols to take into account not only the local state which usually triggers the stimulus, but also a general sensitivity state of the system being modulated in its excitability.

Therefore, this study aims to identify suitable functional pathways between well-known cortical alpha generators, whose connectivity state can be assessed with MEG/EEG at rest. Here, we present a pipeline that effectively determines potential connections from resting MEG/EEG data using Weighted Pairwise Phase Consistency (WPPC; [19]) and Weighted Phase Lag Index (WPLI; [20]). It is worth noticing that the choice of a connectivity metric has to take into account its advantages and disadvantages in the context of application [21,22]. Here we used WPPC and WPLI, exploiting their partial complementarity, for the data under investigation. Both metrics depend on the consistency between the phases of the signal of interest, and are not biased by sample size. WPPC is based on the pairwise phase difference, thus it is still affected by zero-lag correlations introduced by the spatial spread of the inverse solution. As a matter of fact, WPPC also provides positive and statistically significant values when the phase difference is exactly zero. This is a result that does not reflect real synchronization, because the finite (but not null) propagation time of the nervous signal on the pathway connecting the two areas is not taken into account. In contrast, WPLI does not have this problem because it is computed from the sign of the imaginary part of the coherence, which vanishes for zero-lag correlations. However, the WPLI signal to noise ratio is optimal for a phase delay of π/2, which corresponds at a typical frequency of 10 Hz to an absolute time delay of 25 ms. Since we are investigating the temporally resolved cortico-cortical connectivity, this is a large delay if compared to the typical brain conduction delays within the same hemisphere. Thus WPLI, despite being a less sensitive measure of phase consistency between the brain regions under investigation, is useful to confirm that the connectivity already detected by the more sensitive WPPC is real and not due to the spatial spread of the inverse solution. In this sense, the two metrices are partially complementary. With this methodological set-up, we investigated 203 healthy subjects with several minutes of resting MEG data and found alpha connectivity stronger for parietal-prefrontal areas rather than for occipito-parietal areas. The results of this study will be relevant to design real-time brain state-dependent EEG-TMS experiments: connectivity priors [23] will be highly relevant as off-line acquired a priori information for the development of real-time connectivity state estimation algorithms.

## 2. Methods

### 2.1. Dataset and Acquisition

**Figure 1:**
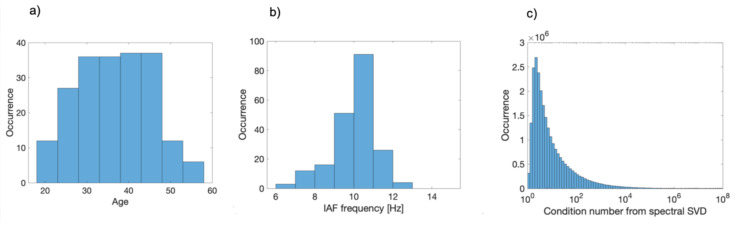
(**a**) Distribution of sample age. (**b**) Distribution of detected IAF on the whole sample. (**c**) Distribution of all condition numbers from SVD, aiming to define optimal dipole direction for spectral data.

We analyzed magnetoencephalographic (MEG) resting state recordings of 203 healthy participants (age range 18–57 years; see Figure 1a for age distribution) from the Cambridge Centre for Aging and Neuroscience (CamCAN) public dataset. Prior to inclusion in the dataset for neuroimaging measurements, all participants were tested for cognitive decline (MMSE > 24; [24]), for matching vision, hearing and English language inclusion criteria and for absence of serious neurological and psychiatric conditions (see [25] for details about the dataset and acquisition parameters). For each participant, resting state activity (eyes closed; 8 min and 40 s) and empty-room noise background (3 min) were recorded using a 306-channel VectorView MEG system (102 magnetometers; 204 first order planar gradiometers; sampling rate = 1000 Hz; high pass filter 0.03 Hz; low pass filter 330 Hz). Anatomical landmarks (nasion, left and right pre-auricular points) were registered, as well as at least 75 additional (isotrak) points, to model the head surface. For human resting state data, continuous monitoring of the head position (cHPI), electro-ocular (EOHG, EOVG) and electro-cardiac (ECG) recordings were available. Additionally, T1 weighted anatomical data were collected using a 3T Siemens TIM Trio scanner (MPRAGE, TR = 2250 ms, TE = 2.99 s; FOV = 256 × 256 × 192; voxel size 1 × 1 × 1 mm). All the subsequent analysis was performed only on planar gradiometers using Fieldtrip ([26]), SPM ([27]), CAT12 (http://www.neuro.uni-jena.de/cat/ (accessed on 1 October 2021)) and custom MATLAB code.

### 2.2. MEG Data Preprocessing

Collected MEG datasets (resting state and empty-room) were inspected and bad channels showing high noise levels and/or SQUID jumps were marked and removed from the data. External magnetic source nuisance was removed by means of temporal Signal Space Separation (tSSS, [28,29]) with a correlation threshold of 0.98 and a sliding time window of 10 s. Line noise contribution was removed by applying a 5th-order Butterworth two-pass filter centered at the line frequency and its first 3 harmonics, and spanning an interval of 2 Hz. Head position of the resting state data was corrected every 200 ms using cHPI data and re-referenced to a common head position. Human MEG data were cleaned from physiological artifacts using an automated procedure. Muscular activity was detected by filtering data between 100 and 140 Hz, transforming each channel data into a z-score relative to the whole channel time series and rejecting segments where the z-score averaged across channels was greater than 5 for at least 200 ms. Then we ran an extended Infomax Independent Component Analysis (ICA; [30,31]) on data filtered between 0.5 and 125 Hz (Butterworth 4th order, two-pass) and resampled to 250 Hz. Data resampling and digital filtering commute with the ICA unmixing matrix estimation, provided the artifactual activity of interest lies in the spared frequency band [31]. For this reason, and for computational efficiency, we computed ICA on resampled data and applied unmixing matrices to unfiltered data at the original sampling rate of 1000 Hz. Correlation coefficients of each extracted component with electro-physiological channels (EHOG, EVOG and ECG) were computed and rejected using a recursive z-score based procedure that robustly discarded all components with correlation more than 2 standard deviations from the coefficient distribution mean of all components. Data cleaned from artifacts were then filtered and segmented. Empty-room data, later used for noise covariance matrix estimation, were filtered broadly between 0.5 and 140 Hz (4th-order Butterworth two-pass filter) and segmented in 1 s epochs. Human resting state recordings were instead filtered around the alpha frequency band (4th-order Butterworth two-pass filter between 5 and 16 Hz) and split into 2 s segments. Finally, all empty-room and resting state segments exceeding a threshold of 10 standard deviations with respect to the channel average, were further discarded in order to deal with potential residual SQUID jumps and/or filter border effects.

### 2.3. Anatomical Data Processing

Structural T1w MRI scans were processed with the aim of extracting cortical surfaces for forward and inverse model calculation and for common space mapping. We processed T1w images using the CAT 12 toolbox pipeline (http://www.neuro.uni-jena.de/cat/ (accessed on 1 October 2021)). After bias normalization, denoising and skull stripping, volumetric data were automatically co-registered to a template space (IXII 555 MNI space; www.brain-development.org, (accessed on 1 October 2021)) Different tissue was then segmented in native space in order to extract surface models of the head, the brain enclosing surface and the cortical mantle. We modeled the cortical mantle with a tessellation (20,484 vertices) of the mid thickness surface, i.e., the surface in between the pial and the white/gray matter interface. The surface enclosing the brain and a surface model of the head were also modeled as 20,000 vertices meshes. In addition to the extracted surfaces in native subject space, co-registered spheres for projection on the FreeSurfer Average (5th order icosahedron “fsaverage5”; [32]) superficial template space were computed, as well as the corresponding interpolation matrices between template and native coordinate spaces. Interpolation coefficients for each point were defined as inversely weighted average of first neighborhood. The mapping of the native space cortical surface onto the FreeSurfer average allowed us to identify, in template space, vertices belonging to 360 regions of interests of the ‘state of the art’ multimodal parcellation from the Human Connectome Project [33]. This atlas was generated by combining structural, diffusion and resting state fMRI data from 210 healthy young adults. Being interested in phase consistency between frontal, parietal and occipital areas, we defined three corresponding brain sectors, for each hemisphere, by pooling together correspondent ROIs from the atlas. We selected the ROIs in order to achieve a sufficiently large coverage of the three sectors of interest, while avoiding excessive proximity that might lead to spurious connectivity due to potential spread of the inverse solution. Finally, this led to 16, 7 and 18 regions of interest for the occipital, parietal and frontal sectors, respectively. For the sake of computational efficiency, we refined the sectors by trimming the borders in order to have the same number of dipoles per sector (D = 930). A list of corresponding regions of interest, with MNI coordinates, can be found in Table 1. All the subsequent analyses were performed only on these ROIs.

### 2.4. MEG Source Reconstruction Based on Individual Anatomies

We used Minimum Norm Estimation [34] to solve the inverse problem and compute the projection matrix from sensors to source space. Native space surfaces were co-registered to MEG coordinates in a first step, using correspondence between anatomical landmarks as recorded during the MEG session and in the MRI anatomical image. Second, co-registration was refined by aligning additional head surface points registered during MEG acquisition to the tessellation representing the head surface. In this procedure, MEG isotrak points anterior to the nasion were discarded since the anatomical MRI images were defaced prior to being publicly available. We then computed a forward model solution for planar gradiometers using the singleshell method and the brain enclosing surface as computed before, and depth normalizing lead fields with a factor of 0.5. The source model for MNE was defined as a free orientation set of dipoles uniformly distributed on the cortical surface (mean spacing 3.1 mm) and the inverse solution was computed, with a 1% noise covariance regularization. This procedure leads to a set of three time series of estimated cortical activations for each resting state epoch and for each vertex of the source model mesh, in the three cartesian coordinate system. MEG is blind to the radial component of magnetic field generated by a current dipole in the source model [34,35]. Thus, even for realistic forward solutions, the estimated current in the most radial direction, with respect to the head surface, is almost zero. Therefore, we performed a Singular Value Decomposition (SVD) of the three source time series at each vertex and for each epoch, retaining only the components associated with the first two singular values, while the third was always almost zero. In this way we reduced the current estimate to its projection on the plane orthogonal to the radial direction, resulting in two source activity time series for each vertex and for each dipole represented hereafter by the two-dimensional vectors ***x****(t)*.

### 2.5. Spectral Analysis

For each epoch and vertex of the cortical mesh, we computed Fourier coefficients ***X****(f)* from the time dependent activity vectors ***x****(t)* in a frequency interval *f* = [8:14] Hz using a Hanning window taper. Having thus two Fourier coefficients at each frequency, dipole and epoch, we decided to reduce spectral data defining an optimal dipole orientation as follows: we computed the cross spectral density matrix between the two Fourier components, performed an SVD and keeping only the direction relative to the first singular value. This resulted in an optimal dipole orientation $\hat{u}$ that depends on the dipole position, the epoch and the frequency of interest, thus optimizing the detection of the brain signal at the frequency of interest. We reduced accordingly the Fourier coefficient vector to a scalar one defined as $X\left(f\right)=X\left(f\right)\cdot \hat{u}$. As a measure of the quality of the procedure, we pooled together all condition numbers resulting from SVDs on all subjects: the average condition number was 1.5 × 10^3^. This means that, on average, the dipole orientations were, in time, almost fixed (see also [36]) and thus our optimization procedure captures most of the spectral content of the data. The distribution of the logarithm of all condition numbers can be inspected in Figure 1c, showing the reliability of the assumption. Fourier coefficients in the optimized direction *X(f)* were then used to compute power spectral density at each vertex and for each epoch. Pooling together all spectral densities and detecting the peak in the frequency band of interest, we defined, for each subject, an Individual Alpha Frequency (IAF); a distribution of all the 203 IAFs is reported in Figure 1b. All the subsequent connectivity analysis was then performed at the individual alpha frequency. For this reason, the frequency dependence of Fourier coefficients *X(f)* will be dropped hereafter in the notation.

### 2.6. Connectivity Analysis and Group Statistical Validation

We are interested in connectivity and phase relationships between region of interest belonging to the frontal, parietal and occipital areas within each hemisphere. For this reason, we computed two different spectral based connectivity metrics between all the combinations of areas belonging to the three different sectors. In particular, given the symmetricity of the connectivity metrics we use, we crossed occipital areas with parietal and frontal, and parietal region of interests with frontal ones. For each resulting combination, we computed the Weighted Pairwise Phase Consistency (WPPC; [20]) and the Weighted Phase Lag Index (WPLI; [19]). The WPPC is based on the distribution of the phase differences between all the pairs of observations (resting state 2 s epochs in our case). WPPC is a robust non-biased measure of phase consistency of brain signals, but it is still affected by zero-lag artificial correlations induced by the imperfection of the inverse model solution [21,22]. Complementary to WPPC, we estimated WPLI as a connectivity measure not affected by the artificial zero-lag correlations. Being based on the imaginary part of the coherence, WPLI is immune to zero-lag connectivity but it has another disadvantage: it achieves maximal Signal to Noise Ratio (SNR) when the two brain signals are in a π/2 relationship. For the frequency band of interest (around 10 Hz) this means a time delay of ~25 ms, a long time when compared to typical brain signal propagation time. However, both metrics strongly depend on a consistent phase relationship between signal of interest, and then they can complementarily provide information about the phase consistency of alpha rhythmicity between the sectors we are investigating.

Given the combination of two brain sectors (*A* and *B*) from the ones defined above (O = occipital; P = parietal; F = frontal) we computed whole sector connectivity matrices, using both WPLI and WPPC, as follows. Named for brevity as $X=X_{A}\left(d_{a}\right)$ and $Y=Y_{B}\left(d_{B}\right),$ the Fourier coefficients at each vertex $d_{a}$ and $d_{b}$ of the sectors *A* and *B*, respectively, we computed a regional dipole-wise connectivity matrix $C_{AB}^{M}\left(d_{a},d_{b}\right)\in M\left(D\times D\right)$, where *M* represents the metrics (M = WPPC or WPLI). Henceforth, for the sake of clarity, subscripts and superscripts will be omitted when not necessary. In addition, we computed, for each metrics, correspondent null connectivity matrices ${\tilde{C}}_{A}\left(d_{a},d_{r}\right)$ and ${\tilde{C}}_{B}\left(d_{b},d_{r}\right)$ from sector of interest to a set of *D* dipoles $\left\{d_{r}\right\}$ randomly chosen within each subject space among the set of dipoles not belonging to any sector of interest. These null connectivity matrices were then used for group statistical validation, under the null hypothesis that connectivity to random dipoles, differently chosen for each subject, will be distributed according only to the metrics’ bias and sensitivity. To this aim we performed a bootstrap procedure at the group level by comparing actual and random connectivity matrices: for each combination (*A*, *B*) and for each subject, the actual connectivity matrix $C_{AB}$ and the random ones ${\tilde{C}}_{A}$ and ${\tilde{C}}_{B}$ were permuted 10,000 times in order to estimate the empirical distribution of the Fisher regularized difference of connectivity $dC\equiv \tan^{-1}\left(C_{AB}\right)-\tan^{-1}\left({\tilde{C}}_{A/B}\right)$. The comparison between the real dC  with its null empirical distribution provided, for each dipole, a bootstrap p-value. (only positive tailed comparisons were considered). Resulting $D^{2}$ *p*-values were then FDR corrected (*q* = 0.05) and only significant elements of C_AB were considered in the subsequent analysis. Furthermore, we reduced the information in each connectivity matrix by summarizing connectivity between the two sets of ROIs $\left\{r_{a}\right\}$ and $\left\{r_{b}\right\}$ from the atlas [33] belonging to sectors *A* and *B,* respectively. To this aim we defined, for all connectivity metrics, the following two quantities:

$$\Delta \left(r_{a},r_{b}\right)\equiv \frac{\#\left[C_{AB}\left(d_{a}\in r_{a},\;d_{b}\in r_{B}\right)\right]}{D}$$

$$\Gamma \left(r_{a},r_{b}\right)\equiv P_{95}\left\{f^{sg}\left[C_{AB}\left(d_{a}\in r_{a},\;d_{b}\in r_{B}\right)\right]\right\}$$

where # [⋅] and f^sg [⋅] represents the count and the distribution of significant connectivity values, respectively, while P_95 [⋅] represents the 95% percentile. The quantities Γ and Δ were computed between regions for each combination {r_a,r_b} and from a single region of interest to all the target sector {r_a,B}. Finally, bi-hemispheric results were collapsed by averaging the contribution of both hemispheres. We defined the quantities Δ and Γ to summarize the connectivity between ROIs and/or sectors, given that inspecting he whole dipole by dipole connectivity matrices would have been confusing and not clear to the reader. The two quantities are conceptually derived from standard graph theory analysis. The connectivity degree Δ(r_a,r_b) simply counts, from the connectivity matrix, the number of statistically significant connections between dipoles of the two ROIs, disregarding the strength of the connectivity and normalizing the count to the total number of dipoles in the sector. This is conceptually analogous to the node degree in the context of network analysis. The mean significant connectivity Γ(r_a,r_b) provides a refinement of the connectivity degree, including the information about the connectivity (or phase consistency) strength. This is achieved by selecting from the connectivity matrix only the statistically significant connections between the dipoles of the two ROIs, extracting the 95% percentile of the resulting distribution instead of just counting them. It is worth noticing that choosing the 95% percentile is a conservative choice, given that usually connectivity values bounded between 0 and 1, even when Fisher regularized, can give rise to non-symmetrical distributions.

## 3. Results

**Figure 2:**
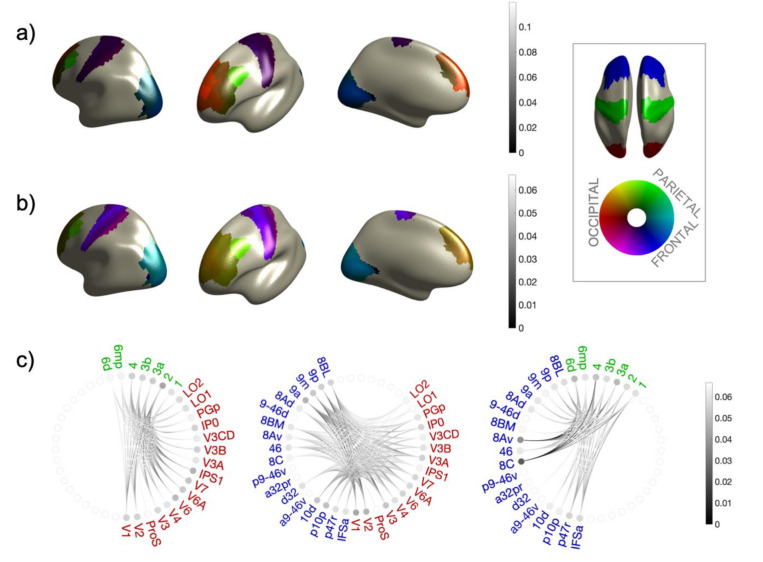
Results for WPPC connectivity. (**a**) Values of $\Delta \left(r_{a},B\right)$, for all sector combinations in color code. (**b**) Values of $\Gamma \left(r_{a},B\right)$, for all sector combinations in color code. (**c**) Connectome plots for the three relevant sector combinations computed from G. Red/green/blue ROI names belongs to occipital/parietal/frontal sectors respectively. The gray level of lines encodes the magnitude of $\Gamma \left(r_{a},r_{b}\right)$ while the gray level of the dot encodes $\Gamma \left(r_{a},B\right)$. In the inset the color code is explained: assigning red/green/blue colors to occipital/parietal/frontal sectors, respectively, the intensity of each area represents the overall magnitude of the parameter of interest, while the color encodes the proportion of the magnitude due to connectivity to the other sectors. So, for example, a parietal area more towards blue has more consistent phase relationship to the frontal sectors, while, if more towards red, the most relevant connection is to the occipital sector.

**Figure 3:**
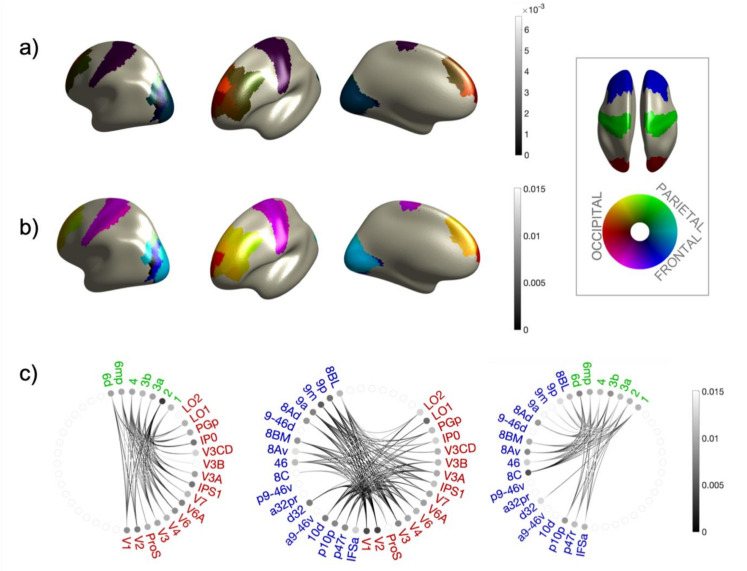
Results for WPLI connectivity. (**a**) Values of $\Delta \left(r_{a},B\right)$ , for all sector combinations in color code. (**b**) Values of $\Gamma \left(r_{a},B\right)$, for all sector combinations in color code. (**c**) Connectome plots for the three relevant sector combinations computed from G. Red/green/blue ROI names belong to occipital/parietal/frontal sectors, respectively. The gray level of lines encodes the magnitude of $\Gamma \left(r_{a},r_{b}\right)$ while the gray level of the dot encodes $\Gamma \left(r_{a},B\right)$. In the inset the color code is explained: assigning red/green/blue colors to occipital/parietal/frontal sectors, respectively, the intensity of each area represents the overall magnitude of the parameter of interest, while the color encodes the proportion of the magnitude due to connectivity to the other sectors. So, for example, a parietal area more towards blue has more consistent phase relationship to the frontal sectors, while, if more towards red, the most relevant connection is to the occipital sector.

Degree and mean connectivity between each ROI combination ($\Delta \left(r_{a},r_{b}\right)$ and $\Gamma \left(r_{a},r_{b}\right)$) and from each ROI to the other sectors as a whole ($\Delta \left(r_{a},B\right)$ and $\Gamma \left(r_{a},B\right)$) for WPPC and WPLI are shown in Figure 2 and Figure 3 as color coded source maps and connecto-grams. All extracted values are listed in Table 2 and Table 3. In general, we can notice that the connectivity estimated from both metrics, each one differently depending on a consistent phase relationship, is predominant in the parietal–frontal connection. While the connection between occipital and frontal sectors appears to be still relevant, the weakest phase consistency has been found between the occipital and the parietal areas. The predominance of phase consistency in parieto–frontal connections is also confirmed by the value of $\Gamma \left(P,F\right)=0.06$, to be compared with $\Gamma \left(O,P\right)=0.03$ and $\Gamma \left(O,F\right)=0.03$ obtained by WPPC considering the whole area as a single ROI in the analysis. When considering the same values but extracted from WPLI, the pattern is less evident ($\Gamma \left(O,P\right)=0.012$; $\Gamma \left(O,F\right)=0.015$ and $\Gamma \left(P,F\right)=0.013$), since the mean connectivity between sectors as detected by the imaginary part of the coherence is almost the same. However, this has to be interpreted in the light of the different sensitivity of the two metrics with respect to the absolute value of phase shift at IAF between the source activity in the two areas, which is ultimately related to the propagation time of the nervous signal on the pathway connecting the ROIs under consideration. As a matter of fact, comparing values of connectivity parameters as extracted from WPPC and WPLI, we can see that the former are in general larger than the first ones. The pattern of a predominant fronto-parietal connectivity with a less consistent pareto-occipital connectivity is also less marked in the WPLI case. We interpret this, methodologically, by considering the different sensitivity of WPPC and WPLI with respect to phase delay absolute values. Being based on the imaginary part of the coherence, WPLI is more sensitive to phase consistent signals whose time delay corresponds to values close to ϕ=π/2, which translate in the alpha band into a propagation time delay of signals of ~25 ms. This time is relatively large if compared to typical brain conduction delays, therefore we expect the phase delay value giving rise to significant connectivity measures to be more toward a phase difference of ϕ=0, thus expecting WPPC to be more sensitive. However, because of the finite conduction delays in the white matter, and in general in the brain, we expect more distant areas having larger propagation time. Thus, we expect phase consistency between distant areas to be more evident in the WPLI.

Inspecting more in detail $\Delta \left(r_{a},B\right)$ and $\Gamma \left(r_{a},B\right)$), as we can see from Figure 2a,b, the overall higher values for both the quantities extracted from WPPC correspond to the pareto–frontal connections, giving frontal area 8C the overall highest values ($\Delta \left(8C,P\right)=0.1$ and $\Gamma \left(8C,P\right)=0.06$)). This predominance can be also appreciated in the connecto-grams (Figure 2c), where connections between frontal areas 8C and 8av appear to be the highest in this sector’s combination and with respect to the other sector combinations. This result is further confirmed by values obtained from WPLI, as shown in Figure 3. In this case also, referring to the parietal-frontal connection, area 8C is the most connected, with $\Delta \left(8C,P\right)=0.0009$ and $\Gamma \left(8C,P\right)=0.013$. We can thus conclude that this pattern is not due to the proximity of area 8C with respect to the frontal sector, WPLI being insensitive to zero-lag connections by design. However, in this case, the degree and the mean connectivity of areas 8c and 8av are not the highest with respect to other sector combinations, as can be appreciated in the connecto-grams of Figure 3c and in the color-coded source maps of mean connectivity in Figure 3b.

As regards the occipito–frontal connections, the highest WPPC connectivity and degree values has been found from frontal area 9a to the occipital sector, being $\Delta \left(9a,O\right)=0.12$ and $\Gamma \left(9a,O\right)=0.03$. The corresponding values for WPLI are $\Delta \left(9a,O\right)=0.003$ and $\Gamma \left(9a,O\right)=0.013$ while, in this case, areas 9m and 9p have also been found among the most connected to the occipital sector ($\Delta \left(9m,O\right)=0.007$ and $\Gamma \left(9m,O\right)=0.015$; $\Delta \left(9p,O\right)=0.007$ and $\Gamma \left(9p,O\right)=0.013$). This pattern is confirmed also by inspecting the connecto-grams in Figure 2c for WPPC and Figure 3c for WPLI, where, in the latter case, connectivity from frontal areas to occipital is more evident than in the WPPC case. As we pointed out before, we interpret this as a byproduct of the different sensitivity of WPPC and WPLI to coherent signals whose phase delay is more towards ϕ=0 or ϕ=π/2.

Finally, also in the single ROI connectivity values inspection, the occipito–parietal connectivity seems to be less relevant. This can be appreciated in the connecto-grams for WPPC in Figure 2c, where the highest values, in this case, can be found in frontal area 2 ($\Gamma \left(2,O\right)=0.034$ and in occipital area V6 ($\Gamma \left(V6,F\right)=0.033$) for the WPPC. This is confirmed also from WPLI results ($\Gamma \left(2,O\right)=0.013$ and ($\Gamma \left(V6,F\right)=0.012$). However, in this case the predominance of these two areas in the occipito-parietal connectivity is less marked as can be noticed from the connecto-grams in Figure 3c.

## 4. Discussion

Our aim was to provide the brain-state dependent EEG-TMS community with a work describing functional phase-dependent relationships between frontal, parietal and occipital areas. In these regions, alpha phase-dependent cortical excitability has been studied both in healthy controls and patients via brain-state dependent stimulation [8,13,14,15]. (Since the attempts at manipulating the effects of stimulation triggered by the phase detected on a region different from the target have no clear effectiveness [8], we believe that taking into account off-line phase-dependent connectivity patterns between the areas of interest would be of substantial help in predicting results from these studies.

Furthermore, consideration of the general phase-dependent connectivity state of the target region, (either trigger-region or not), could be essential for explaining changes in connectivity patterns for the time after application of plasticity protocols up to the moment where the behavioral measure has returned to baseline. Considering not only the TMS trigger-state (phase of the frequency of interest) but also the general connectivity state of the brain between the regions of interest would in fact be useful both for predicting plasticity protocols’ efficacy and for interpreting results. For these reasons, we investigated spectral-resolved connectivity metrics that depend on a consistent phase relationship at IAF between frontal, parietal and occipital regions at rest. Results showed greater functional coupling between frontal and parietal areas, compared to very low functional coupling values between frontal and occipital and parietal and occipital pairs (Figure 2). These results are consistent with previous evidence of a fronto-parietal network at rest (e.g., [37]). We used MEG data and three different phase-coherence measures to show that a clear coupling emerges at IAF between frontal and parietal parcels at rest.

The ability to assess the strength of frontoparietal coupling from resting state data before and after an intervention makes this a suitable pathway for the investigation of pathway-specific plasticity. The alpha frequency band is relevant because this is the frequency band where phase-specific modulation of cortical excitability was previously demonstrated both in the motor system [8,13,14] and in the prefrontal cortex [15]. Therefore, in frontal and sensorimotor regions, state-dependent stimulation based on the phase of alpha frequency appears effective in modulating cortical excitability at rest. However, when TMS is applied to the motor cortex in response to the phase of alpha recorded from the occipital cortex at rest, no modulation of corticospinal excitability was found [8].

For future development of real-time estimators of instantaneous connectivity states, we propose that connectivity metrics which depend on phase relationship between areas, and therefore describe whether two regions share trial-averaged functional coupling, can provide off-line connectivity priors in order to enable more accurate time-resolved connectivity estimates. This broad state of functional coupling will, in fact, constitute a novel level of a priori information to assess whether real-time EEG-TMS paradigms will be effective in modulating cortical excitability in one area when the trigger is recorded from another area. In this light, for example, our finding of the limited functional coupling at rest between occipital and parietal areas can help elaborate on the reason why no effect was found when the hand knob of the motor cortex was stimulated on the basis of the occipital alpha phase [8].

Therefore, we propose that, in order to improve the efficacy of brain-state dependent stimulation protocols, the definition of “brain state” should not only refer to the instantaneous phase of the considered frequency which is triggering the stimulus, but also to the predisposition of the network to be globally perturbed by a stimulus triggered by a given phase of a determined frequency. In this regard, measures of functional coupling of regions at rest or during tasks that involve different functional coupling patterns would allow the obtaining of “off-line” connectivity priors.

In essence, beside the targeting of a local oscillation phase, brain-state dependent stimulation might leverage the off-line acquired, a priori knowledge regarding functional patterns in order to causally test the functional coupling between the brain regions involved. As an example of potential relevance of this approach, it has been shown that a network of prefrontal and occipito–parietal areas is involved in visual target detection and that alpha phase in this network is essential for a correct visuospatial processing [38,39]. Standard brain-state dependent stimulation could be used in order to further test the relationship between these cortical areas. In fact, brain-state dependent stimulation could potentially modulate cortical excitability of one area given the phase of alpha from another region. However, according to our approach, this brain-state dependent stimulation could lead to more effective results only when the two regions are embedded in a functional pattern that describes a state of sensitivity to be modulated [13,21]. A relevant part of cortical excitability could derive therefore from functional coupling of the target areas at rest (or during a task) at a given frequency.

This new perspective would also be useful for developing new and more effective individualized approaches for rehabilitation via brain-state dependent stimulation. In this regard, individualized stimulation rehabilitative protocols have been proposed in stroke patients for whom specific adaptive connectivity patterns between specific cortical regions should be reinforced in order to achieve recovery [40]. In this light, our approach combining brain-state dependent stimulation with phase-dependent connectivity measures would nicely suit this purpose.

Finally, it must be noticed that our work is the first attempt at measuring coherence at IAF during rest between these three regions of the cerebral cortex. Most of the resting state literature consists of fMRI studies, which lack the temporal resolution that MEG measures provide [41,42,43]. It is also worth noticing that, depending on the connectivity metric employed, we obtain slightly different results in the coupling between frontal and occipital areas, with the WPLI showing some functional coupling between the two regions compared to other measures. The WPLI is a measure based on the imaginary part of coherence. This metric is not sensitive to zero-lag spurious correlations. However, its maximum SNR spuriously coincides with π/4. Since we are investigating IAF which, on average, refers to around 10 Hz, the maximum SNR of WPLI measures has a phase-lag of about 25 ms. For these reasons, the functional relationship between distant areas like the frontal and occipital cortices might be captured thanks to this longer phase-lag that allows pick-up of the coupling between signals with longer propagation time.

To summarize, our findings suggest that brain-state dependent stimulation could benefit from taking into account a broader concept of “state” of the system. Depending on whether brain-state dependent stimulation is practiced at rest or during a task, one should consider the functional coupling patterns between the areas of interest in the frequency band whose phase is used as a trigger. Furthermore, we suggest that the physical distance between the regions could also be taken into account when choosing the coherence metric to assess functional coupling based on a consistent phase relationship in the frequency band of interest.

## 5. Conclusions

Brain-state dependent brain-stimulation has the potential to reliably modulate specific neural pathways using a “Closed-Loop” approach [44]. How to achieve a time-resolved real-time estimation of EEG-derived brain connectivity states remains a key challenge. In this study, we addressed the off-line estimation of phase-based connectivity patterns between different regions of interest, presenting a pipeline to determine candidate pathways for real-time paradigms, for instance where the trigger-signal is extracted from one cortical site, and the stimulation targets a different site, or considering the target region and downstream effects to distal cortical regions.

In summary, offline connectivity measures have a twofold purpose: first, the estimate of connectivity patterns before and after can represent a biomarker of cortico-cortical changes after single and repetitive state-dependent TMS. In fact, for mu-alpha, stimulation at troughs was found to enhance Transcranial evoked potentials (TEPs) in both ipsi and contralateral hemisphere at 100 ms after stimulus even when the stimulus was provided at 90% of motor threshold [10]. Moreover, the same kind of stimulation has been found to be responsible for an enhancement of connectivity between the two sensorimotor cortices [45]. Connectivity joined with TEP analysis could be even more relevant when analyzing non-motor areas such as the dorsolateral prefrontal cortex, where one cannot rely on as reliable and consistent an outcome as that of the MEPs.

Second, an off-line connectivity analysis can be used as prior information for future online estimates, to determine a correlation between areas oscillating at the same frequency or in an anticorrelation, a phase state which was previously suggested as a possible marker of functional segregation [46], or no correlation at all. The offline measures can serve as a benchmark for determining the optimal trade-off between the temporal resolution (window-length) and the estimator error variance for a given experimental condition, noting that the window-length must be short enough to capture physiological transitions [47].

## Figures and Tables

**Table 1:** List of Region of Interest (ROI) defining the three sectors. A brief description (from van Essen) and the coordinate of the centroid in mm with respect to the template MNI space is reported.

ROI	Sector	MNI Coordinates of Centroid (mm)		
		x	y	z
**V1**	Occipital	−13.1	−82.0	1.5
**V2**	Occipital	−12.4	−81.5	3.6
**ProS**	Occipital	−18.5	−52.2	0.1
**V3**	Occipital	−18.3	−86.2	5.4
**V4**	Occipital	−29.7	−82.5	−3.9
**V6**	Occipital	−13.9	−78.0	27.2
**V6A**	Occipital	−18.6	−84.3	38.1
**V7**	Occipital	−23.8	−81.9	26.6
**IPS1**	Occipital	−22.6	−71.7	33.0
**V3A**	Occipital	−17.2	−88.4	23.0
**V3B**	Occipital	−28.2	−78.9	16.3
**V3CD**	Occipital	−35.3	−85.7	12.3
**IP0**	Occipital	−30.4	−73.5	25.5
**PGp**	Occipital	−39.8	−80.1	22.1
**LO1**	Occipital	−37.8	−82.9	4.2
**LO2**	Occipital	−42.7	−83.3	−4.9
**1**	Parietal	−47.1	−24.5	52.3
**2**	Parietal	−35.4	−34.4	49.7
**3a**	Parietal	−34.3	−21.8	41.8
**3b**	Parietal	−36.8	−24.1	51.6
**4**	Parietal	−26.7	−19.7	53.8
**6mp**	Parietal	−14.1	−13.2	65.7
**6d**	Parietal	−34.9	−12.7	61.9
**8BL**	Frontal	−11.6	35.1	50.8
**9p**	Frontal	−18.9	44.0	36.4
**9m**	Frontal	−7.7	51.0	21.8
**9a**	Frontal	−19.7	53.2	23.8
**8Ad**	Frontal	−23.3	24.7	41.2
**9** **–** **46d**	Frontal	−28.7	42.1	21.4
**8BM**	Frontal	−6.3	29.5	43.1
**8Av**	Frontal	−37.1	18.0	47.4
**46**	Frontal	−36.6	35.6	28.3
**8C**	Frontal	−40.3	16.1	35.0
**p9** **–** **46v**	Frontal	−43.3	29.2	26.3
**a32pr**	Frontal	−10.2	28.1	28.6
**d32**	Frontal	−10.0	38.5	21.1
**a9** **–** **46v**	Frontal	−37.1	47.7	8.8
**10d**	Frontal	−12.1	62.9	8.4
**p10p**	Frontal	−23.6	55.0	5.2
**p47r**	Frontal	−41.2	40.3	1.5
**IFSa**	Frontal	−42.0	31.2	13.2

**Table 2:** Connectivity values for all ROIS towards each of the three sectors. Values of $\Gamma \left(r_{a},b\right)$ and $\Delta \left(r,B\right)$ computed from WPPC are shown.

	Occipital		Parietal		Frontal	
**V1**			0.00005	0.01813	0.04801	0.03330
**V2**			0.00026	0.02336	0.03238	0.03052
**ProS**						
**V3**			0.00054	0.02260	0.01536	0.02715
**V4**			0.00007	0.01750	0.00167	0.01769
**V6**			0.01140	0.03235	0.01202	0.02595
**V6A**			0.00591	0.02071	0.01167	0.01895
**V7**			0.00787	0.02024	0.01866	0.02350
**IPS1**			0.01689	0.03146	0.00290	0.01733
**V3A**			0.00240	0.02260	0.01683	0.02353
**V3B**			0.00526	0.02268	0.00203	0.01546
**V3CD**			0.00017	0.01639	0.00426	0.01660
**IP0**			0.00256	0.02089	0.00459	0.01869
**PGp**			0.00036	0.01662	0.00583	0.01964
**LO1**					0.00054	0.01236
**LO2**			0.00007	0.01632	0.00008	0.01108
**1**	0.00147	0.01513			0.01000	0.02590
**2**	0.01323	0.03352			0.00150	0.02274
**3a**	0.00182	0.02163			0.01169	0.06285
**3b**	0.00166	0.01980			0.00928	0.04439
**4**	0.00153	0.01990			0.01354	0.06664
**6mp**	0.00026	0.01459			0.00653	0.03106
**6d**	0.00037	0.01261			0.02895	0.05629
**8BL**	0.09001	0.02671	0.00231	0.01952		
**9p**	0.08669	0.02861	0.00182	0.01623		
**9m**	0.10542	0.03381	0.00006	0.01323		
**9a**	0.11954	0.02970	0.00015	0.01537		
**8Ad**	0.03460	0.01914	0.00374	0.01783		
**9–46d**	0.05946	0.02154	0.00038	0.01336		
**8BM**	0.04143	0.02319	0.00015	0.01470		
**8Av**	0.00697	0.01461	0.06058	0.04594		
**46**	0.01625	0.01679	0.00248	0.01423		
**8C**	0.00071	0.01432	0.10138	0.05798		
**p9–46v**	0.00158	0.01213	0.01557	0.01848		
**a32pr**	0.00488	0.01591	0.00003	0.00649		
**d32**	0.02711	0.02249	0.00003	0.00840		
**a9–46v**	0.04548	0.01925	0.00283	0.01968		
**10d**	0.07100	0.02592	0.00028	0.01980		
**p10p**	0.05559	0.02151	0.00038	0.01635		
**p47r**	0.01739	0.01740	0.01290	0.02046		
**IFSa**	0.00425	0.01535	0.00776	0.01874		

**Table 3:** Connectivity values for all ROIS towards each of the three sectors. Values of $\Gamma \left(r_{a},b\right)$ and $\Delta \left(r,B\right)$ computed from WPLI are shown.

	Occipital		Parietal		Frontal	
**V1**			0.00004	0.01007	0.00103	0.01402
**V2**			0.00002	0.01189	0.00103	0.01511
**ProS**					0.00008	0.00814
**V3**			0.00004	0.01173	0.00041	0.01337
**V4**					0.00012	0.01177
**V6**			0.00007	0.01102	0.00013	0.01063
**V6A**			0.00014	0.00900	0.00013	0.01001
**V7**					0.00019	0.01040
**IPS1**			0.00023	0.01053		
**V3A**			0.00005	0.01201	0.00014	0.01081
**V3B**					0.00016	0.01120
**V3CD**						
**IP0**			0.00016	0.01141	0.00003	0.00919
**PGp**			0.00009	0.01005	0.00009	0.01133
**LO1**						
**LO2**					0.00017	0.00917
**1**	0.00010	0.00946			0.00006	0.01010
**2**	0.00022	0.01309			0.00006	0.01011
**3a**	0.00008	0.01093			0.00022	0.01174
**3b**	0.00013	0.01032			0.00011	0.01131
**4**	0.00015	0.01078			0.00017	0.01302
**6mp**	0.00007	0.00970			0.00009	0.00931
**6d**	0.00018	0.01071			0.00027	0.01096
**8BL**	0.00266	0.01300	0.00008	0.01005		
**9p**	0.00664	0.01340	0.00006	0.00831		
**9m**	0.00505	0.01418	0.00003	0.00788		
**9a**	0.00261	0.01296				
**8Ad**	0.00063	0.01018	0.00010	0.00953		
**9–46d**	0.00206	0.01211	0.00002	0.00974		
**8BM**	0.00133	0.01180	0.00002	0.00744		
**8Av**	0.00011	0.00886	0.00074	0.01142		
**46**	0.00043	0.01072	0.00010	0.00949		
**8C**	0.00003	0.00789	0.00090	0.01296		
**p9–46v**	0.00017	0.00939	0.00025	0.01076		
**a32pr**	0.00051	0.01047	0.00002	0.00623		
**d32**	0.00134	0.01224	0.00003	0.00763		
**a9–46v**	0.00119	0.01163	0.00005	0.00786		
**10d**	0.00269	0.01193				
**p10p**	0.00150	0.01111	0.00012	0.00804		
**p47r**	0.00051	0.01068	0.00007	0.00797		
**IFSa**	0.00029	0.00949	0.00011	0.00918		

## Data Availability

Data analyzed in this study are part of the Cambridge Centre for Aging and Neuroscience (CamCAN) public dataset [25] and are publicly available.
